# Invasion front dynamics of interactive populations in environments with barriers

**DOI:** 10.1038/s41598-022-04806-x

**Published:** 2022-01-17

**Authors:** Youness Azimzade

**Affiliations:** Department of Physics, University of Tehran, Tehran, 14395-547 Iran

**Keywords:** Invasive species, Tumour heterogeneity, Computational models, Scale invariance, Biophysics, Evolution, Physics, Biological physics, Statistical physics, thermodynamics and nonlinear dynamics

## Abstract

Invading populations normally comprise different subpopulations that interact while trying to overcome existing barriers against their way to occupy new areas. However, the majority of studies so far only consider single or multiple population invasion into areas where there is no resistance against the invasion. Here, we developed a model to study how cooperative/competitive populations invade in the presence of a physical barrier that should be degraded during the invasion. For one dimensional (1D) environment, we found that a Langevin equation as $dX/dt=V_ft+\sqrt{D_f}\eta (t)$ describing invasion front position. We then obtained how $V_f$ and $D_f$ depend on population interactions and environmental barrier intensity. In two dimensional (2D) environment, for the average interface position movements we found a Langevin equation as $dH/dt=V_Ht+\sqrt{D_H}\eta (t)$. Similar to the 1D case, we calculate how $V_H$ and $D_H$ respond to population interaction and environmental barrier intensity. Finally, the study of invasion front morphology through dynamic scaling analysis showed that growth exponent, $\beta$, depends on both population interaction and environmental barrier intensity. Saturated interface width, $W_{sat}$, versus width of the 2D environment (*L*) also exhibits scaling behavior. Our findings show revealed that competition among subpopulations leads to more rough invasion fronts. Considering the wide range of shreds of evidence for clonal diversity in cancer cell populations, our findings suggest that interactions between such diverse populations can potentially participate in the irregularities of tumor border.

## Introduction

Invasion is a generic process that emerges across different scales and populations^1,2^. During the invasion, new, possibly fitter species occupy further areas mostly at the expense of extinction of existing populations, putting the existing populations in danger. As such, understanding how invasion happens and what related parameters regulate it is of great interest across different fields^3–5^. The invasion has been under investigation for about a century by mathematicians and physicists^6^. Yet, many questions remained to be tackled, particularly where invasion and evolutionary processes are interrelated^7^.

When tumor cells invade into surrounding tissues, invasion becomes a concerning health threat. Thus, understanding the tumor invasion is not only of theoretical interest, but it also can reveal driving mechanisms behind aggressive behavior^8^. In invasive tumors, cancer cells take over the host tissue by pushing existing healthy cells^9^ and degrading the physical structure of extracellular matrix (ECM)^10–12^ alongside various chemical and mechanical interactions^13,14^. Facing such a barrier can affect the evolutionary dynamics of tumors in different aspects^15,16^. More importantly, tumor cells that push the healthy tissue belong to different clones^17^. These subpopulations may cooperate^18–21^ or compete^22–24^ with each other during their way to invade the surrounding healthy tissue^25^. Despite huge literature on clonal diversity in tumors, it is not clear that how such interactions regulate invasion and how the intensity of environmental barriers restricts invasion.

For the invasion that emerges as a result of consecutive duplication and migration of species, one can write^6,26^ ${\dot{C}}=R(C)C+\nabla (D\nabla C)$ where *C*, *R* and *D* represent population density, duplication rate and diffusion constant, respectively. Such a model predicts that invasion happens through traveling Fisher’s waves with velocity of $V_f=2\sqrt{RD}$. Adding number fluctuations to this model leads to fluctuations in propagating waves. For most cases, a Langevin equation provides appropriate representation for these invasion fronts. Thus, for invasion front, *X*, one can write $dX/dt=V_f t+\sqrt{D_f}\eta (t)$ where $\eta$ is noise and $\langle \eta (t)\eta (t')\rangle =\delta (t-t')$^27–29^. Such analysis suggests that by finding $V_f$ and $D_f$ one can describe invasion at least in 1D environments. In two or higher dimensions, invasion fronts can exhibit additional features such as roughness that can provide additional information too^30,31^.

The geometry of tumor cells’ invasion front has been studied from different perspectives. Part of this interest originated from the observation that the geometry of the invasion front is associated with adverse outcomes such as shorter survival time^32,33^. However, it is not clear how the geometry of the invasion front participates in poor clinical outcomes. On the other hand, the notion that invasion front geometry might reveal the driving mechanism behind the invasion^34,35^ has sparked various studies on scaling properties of cancer cells invasion front in vivo^36–38^ and using different mathematical models^39–43^. Despite the development of a diverse range of models on tumor invasion, clonal interaction remained overlooked.

Motivated by interactions for cancer cells and inspired by a model on cooperative populations in the presence of environmental barriers^44^, we developed a model to study how environmental stress regulates invasion front of interactive species. For the 1D case, we tried to see whether any Langevin equation, as predicted by stochastic reaction-diffusion studies, does describe invasion front movements and then obtained corresponding dependencies on environmental stress for cooperative/competitive populations. For the 2D environment, after finding the Langevin equation of invasion front motion, we considered it a growing interface and studied how scaling exponents depend on environmental stress and inter-specific interactions.

## Model

**Figure 1:**
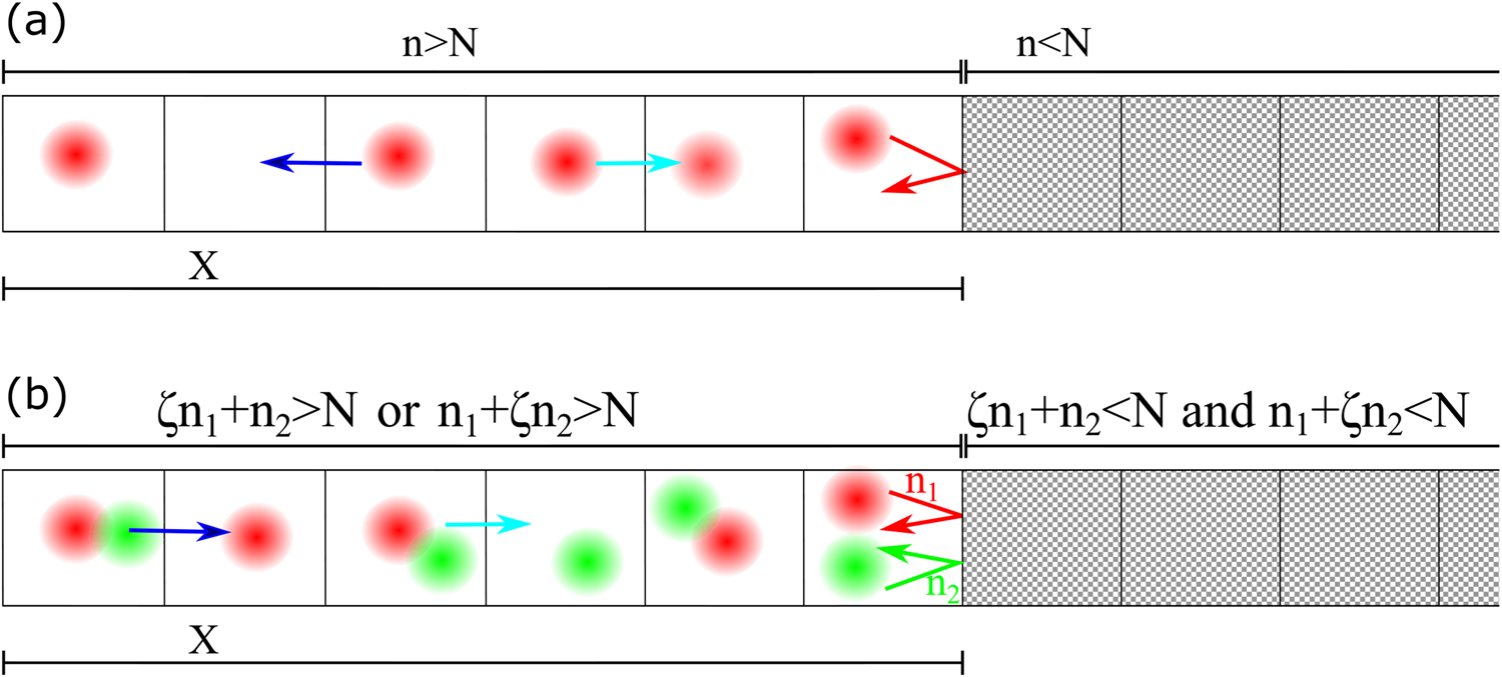
(**a**) Schematic illustration of the single-species model in one dimension. A unit randomly will be selected and duplication and migration trial happen independently. If the selected unit contains a species and two nearest neighbors are empty, the species duplicates into one and can migrate to the other one. The blue arrow shows the direction of upcoming migration to an empty nearest neighbor and the cyan arrow shows upcoming duplication into an empty nearest neighbor. The red arrow shows a failed trial to occupy an empty nearest neighbor. While this attempt has failed, the strength of the barrier has decreased by 1. In a simple single-species model, after $n=N$ trials, the unit becomes occupiable. (**b**) Schematic illustration of the two-species model in one dimension. In a randomly selected unit, each species tries to occupy an empty nearest neighbor. We should have $\zeta n_1+n_2>N$ or $n_1+\zeta n_2>N$ for a unit to become occupiable. Migration and duplication happen similar to the single-species model. If the selected unit contains both species, one will be selected randomly for duplication (migration) first and then the other will be selected. However, since we do not include spatial exclusion, selection does not have a relevant role in the majority of cases.

Here, we develop an individual-based model in which species live on lattice units. The single-species model in a 1D environment follows these rules: As the initial condition, one cell is located at the first unit. For time evolution, a unit will be selected randomly. Throughout this work, one time step is counted when the number of random selections reaches the number of units defined in the model. If the selected unit does contain a species and there is an empty nearest neighbor (NN) (if the same species occupy both NNs, unit selection will be repeated), then (i) it decides to duplicate into an empty NN and would do so if that unit is occupiable. If the selected NN is not occupiable, the trial number for that NN, *n*, increases by one (barrier intensity decreases by one). (ii) Independent of the duplication process, the species decides to migrate to an empty NN and do so if the selected NN is occupiable. If the selected NN is not occupiable, the trial number for that unit, *n*, increases by one (barrier intensity decreases by one). (iii) Any unit would be occupiable after $n\ge N$ times being selected for migration or duplication where *N* is environmental barrier intensity (see Fig. 1a). Based on these rules, if a species lives in a unit with two empty and occupiable NNs, each one of them can be occupied by newly created species or through migration. First, the species decides to duplicate and choose one of the NNs to duplicate into it. Since both NNs are empty and occupiable, one of them randomly will be occupied by the species. Then, the initial species decides to migrate. One of the NNs will be selected and if the empty one gets elected, migration happens. If the filled unit is set for migration, the trial fails.

In the two-species model, for simplicity, we consider species to be able to occupy a unit simultaneously. Due to this assumption, they do not compete for space and interaction between species is limited to their mutual effort to degrade the environmental barrier at invasion front^44^. As the initial condition, the first unit is occupied by two species. For time evolution, a unit is selected randomly. If the unit contains one species, it will evolve based on the rules mentioned above (i–iii). If the unit contains both species, one will be selected randomly for duplication and then the other one will be selected for duplication. For migration, again, one of the species will be selected to migrate first. In their interactions with the environment, we count their trials separately as $n_1$ and $n_2$. For an entirely cooperative scenario, if the number of trials on a unit together exceeds the barrier intensity, $n_1+n_2>N$, the unit becomes occupiable. In a more complex scenario, a unit would be occupiable if we have $\zeta n_1+n_2>N$ or $n_1+\zeta n_2>N$ in which $\zeta$ is the interaction parameter. When two populations are cooperative (competitive) we have $\zeta >0$ ($\zeta <0$). We anticipate a Langevin equation for invasion front, *X* (see Fig. 1b), and we would try to find out how the diffusion constant and velocity of this interface is related to environmental barrier intensity and interspecific interactions. The 2D version of the model, which is an extension of 1D, will be explained later.

## Results

### 1D case

**Figure 2:**
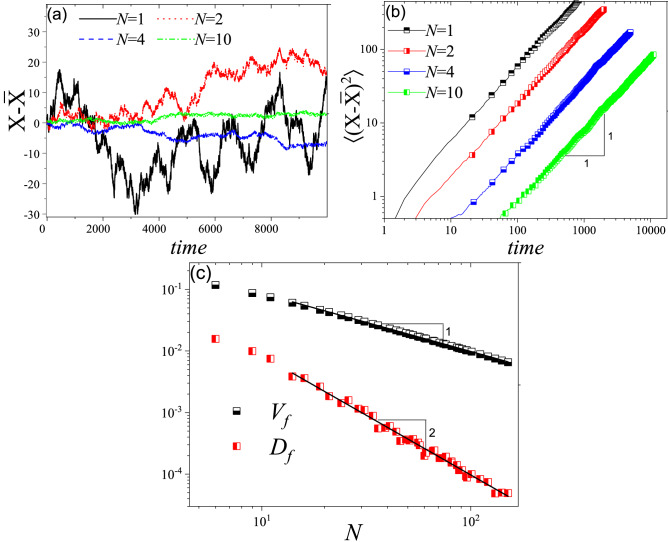
(**a**) Realization of $X-{\bar{X}}$ versus time for different values of *N* for the single-species model. (**b**) $\langle (X-{\bar{X}})^{2}\rangle$ versus time for different values of *N*. The linear behavior in *log*/*log* diagram and the slope of one ensures the random walk like behavior of fluctuations and thus we can write: $\langle (X-{\bar{X}})^{2}\rangle =D_ft$. (**c**) Invasion front velocity and diffusion constant versus environmental barrier intensity, *N*. For the large values of *N*, we have $V_f\propto N^{-\gamma _V}$ with $\gamma _V=1\pm 0.05$ and $D_f \propto N^{-\gamma _D}$ with $\gamma _D=2\pm 0.05$.

First, we study the one-dimensional case. We locate a cell at the first unit of a half limited array and let the system evolve based on the above-mentioned rules. We call the occupied unit with the largest distance from the origin as the invasion front (border) location and call its index as *X*. To find the behavior of invasion front and quantify it, we analyze *X*, ${\bar{X}}$ and $X-{\bar{X}}$ where ${\bar{X}}$ is the ensemble average of *X* over different realizations. Analysis of $X-{\bar{X}}$ versus time (Fig. 2a, b) shows that while *N* affects the magnitude of fluctuations for $X-{\bar{X}}$, the mean squared displacement behaves like a simple random walk and we have: $\langle (X-{\bar{X}})^{2}\rangle \sim t$. As a result, we can define a diffusion constant for these fluctuations as $\langle (X-{\bar{X}})^{2}\rangle =D_f t$. Then we studied the dependency of $D_f$ on *N*. It appeared that for large values of *N*, we have $D_f\propto N^{-\gamma _D}$ with $\gamma _D =2.00 \pm 0.05$ (Fig. 2c). The averaged velocity of the front position gives us the invasion velocity, $V_f$. Invasion velocity also depends on *N* as $V_f\propto N^{-\gamma _V}$ with $\gamma _V =1.00 \pm 0.05$ (Fig. 2c). Such an effect on invasion velocity is expected from an analytical perspective. In a simple 1D invasion model, invasion front velocity, assuming no migration, should be proportional to duplication rate (which is different from that of a fisher’s equation, $V_f\propto \sqrt{RD}$). Since duplication happens after *N* trials, considering the environmental barriers slows down the duplication rate by the factor of *N*. Respectively, invasion velocity should be slower by the same factor ($N^{-1}$). Regarding the dependency of the diffusion constant on *N*, it is enough to look at the definition of $D_f$. As mentioned, invasion velocity decreases by the factor of *N*. Thus, we have $D_f \propto \langle (X/N-{\bar{X}}/N)^{2}\rangle$ which leads to $D_f \propto N^{-2}$.

**Figure 3:**
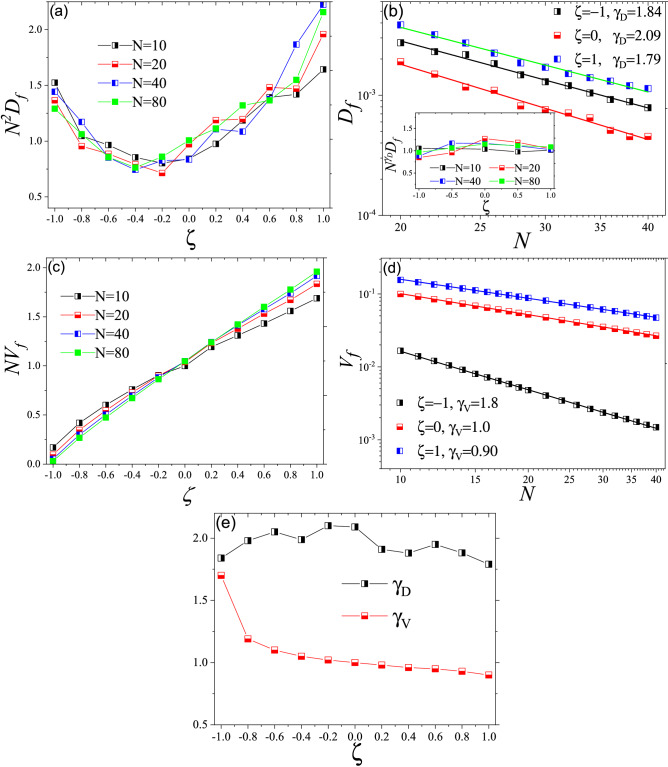
(**a**) The effect of interaction term, $\zeta$ on normalized diffusion constant, $N^{2}D_f$ for different values of *N*. As it shows, interaction term affects diffusion constant differently. (**b**) $D_f$ versus *N* for different interactions. Interestingly, $\gamma _D$ depends on $\zeta$. Inset shows $N^{\gamma _D}D_f$ versus $\zeta$ for different values of *N*. (**c**) Normalized invasion velocity versus $\zeta$ for different values of *N*. (**d**) *V* versus *N* for different interactions. $\gamma _V$ also depends on $\zeta$. Based on this figure, competitive populations are more sensitive to environmental stresses. (**e**) $\gamma _D$ and $\gamma _V$ versus $\zeta$. While $\gamma _V$ monotonically decreases by $\zeta$, $\gamma _D$ has the maximum at $\zeta \sim 0$.

We now add a second population which does not interfere with the first population except for degrading the barrier in the invasion front. As such, the two populations see each other only on the invasion front. We start the model with two species, located at the first unit and use the same definition for the border, but it does not matter which population has occupied that unit. As mentioned, a unit would be occupiable only if $\zeta n_1+ n_2>N$ or $n_1+\zeta n_2>N$. The positive values of $\zeta$ show the cooperation between entities and negative values represent the competitive populations. We first try to see how the normalized diffusion constant, $N^{2}D_f$, depends on $\zeta$. As Fig. 3a shows, the interaction changes the diffusion constant. Both competitive ($\zeta <0$) and cooperative ($\zeta <0$) populations have higher diffusion constant in respect to non-interactive populations ($\zeta =0$). To see how interaction affects the system response to *N*, we study the behavior of $D_f$ versus *N* for different interactions (Fig. 3b). Interestingly, the magnitude of diffusion constant depends on interactions, but its behavior versus *N*, exhibited in value of $\gamma _D$, depends on interactions. As such, interaction leads to higher diffusion constant with smaller $\gamma _D$.

We also studied the effect of interactions on invasion velocity, $V_f$. As Fig. 3c shows, cooperation (competition) increases (decrease) the invasion velocity but the effect also is intensified by *N*. Analysis of behavior of $V_f$ versus *N* shows that $\gamma _V$ also depends on $\zeta$ (Fig. 3d). Finally, as Fig. 3e shows, $\gamma _V$ and $\gamma _D$ differently depend on interaction term, $\zeta$. 1D environment may not seem realistic, yet it has played a central role in advancing our understanding of invasion^6,29^. 1D version of our model reveals that competition changes invasion velocity but, for a wide range of interactions ($-0.6< \zeta < 1$), invasion properties remain primarily unchanged, suggesting that even competition may not affect the invasion velocity significantly. However, to better understand invasion, we need to study the problem in higher dimensions.

### 2D case

**Figure 4:**
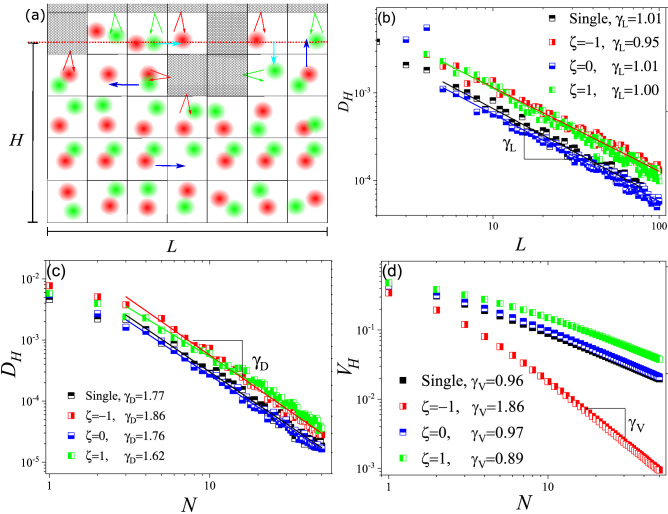
(**a**) Schematic illustration of the two-species model in 2D. All arrows represent the same process as their 1D counterparts. (**b**) $D_H$ versus *L* for different values of $\zeta$ compared to the single population model. As one may expect, $D_H$ behaves as $\sim L^{-1}$ for all interactions similarly. (**c**) $D_H$ versus *N* for $L=20$ and different values of $\zeta$ and single population model. (**d**) Average velocity of interface, $V_H$, versus *N*.

We studied the two-dimension version of our model as well. The 2D version is essentially a semi-infinite array of sites in one direction (just like the 1D case) and a periodic array of sites in the perpendicular direction, with *L* sites. The same rules will be applied to the 2D case (see Fig. 4a). As the initial condition, all units in the first row will be occupied by species. Migration and duplication can happen into four NNs around each randomly selected unit in single-species and two-species cases. We analyze two different aspects of invasion in 2D environments: invasion velocity and the geometry of the invasion front. The average location of interface, *H*, is considered as the location of invasion front by setting $H={\bar{X}}$ in which ${\bar{X}}$ stands for the average value of *X* along the border. Similar to 1D, we anticipate a Langevin equation as $dH/dt=V_Ht+\sqrt{D_H}\eta (t)$ to govern the temporal evolution of *H*. We analyzed ${\bar{H}}$ and $H-{\bar{H}}$ in which ${\bar{H}}$ is the ensemble average. $H-{\bar{H}}$ fluctuates over time like a random walker and we have: $\langle (H-{\bar{H}})^{2}\rangle =D_Ht$. Since *H* is averaged over *L* points (more accurately, $L^{D'}$ in which $D'$ is the fractal dimension of interface), we anticipate fluctuations of $H-{\bar{H}}$ to be scaled as $1/ \sqrt{L}$. As Fig. 4b shows, $D_H\sim L^{-\gamma _L}$ with $\gamma _L\simeq 1$ for all values of $\zeta$. Effect of *N* on $D_H$ was studied and as Fig. 4c shows, $\gamma _D$ slightly decreases as we increase $\zeta$ and we have $\gamma _D=1.86 \pm 0.03$, $\gamma _D=1.86 \pm 0.03$, $\gamma _D=1.76 \pm 0.03$ and $\gamma _D=1.62 \pm 0.05$ for $\zeta =-1$, $\zeta =0$ and $\zeta =1$ respectively.

Then we studied how ${\bar{H}}$ evolve during time to find the interface velocity, $V_H$. As Fig. 4d shows, $V_H$ depends on *N* as $V_f$ did.

Due to importance of the geometry of invasion front, we study of the morphology of invasion front through dynamic scaling analysis. For such analysis, we need to calculate surface’s width as $W^2=\frac{1}{L}\sum _i^L(X_i-H)^2\;$ where $X_i$ is the invasion front at point *i*. For variety of surfaces that follow scaling, one has $W\approx L^\alpha f(t/L^z)$ where *f*(*u*) is a scaling function such that, $f(u)\propto u^\beta$ if $u\ll 1$, and $f(u)\approx$ constant for $u\gg 1$, so that for a fixed *L*, $W\propto t^\beta$. $\alpha$ and $\beta$ are, respectively, the surface roughness and growth exponents, and $z=\alpha /\beta$ is the dynamic exponent^45,46^.

**Figure 5:**
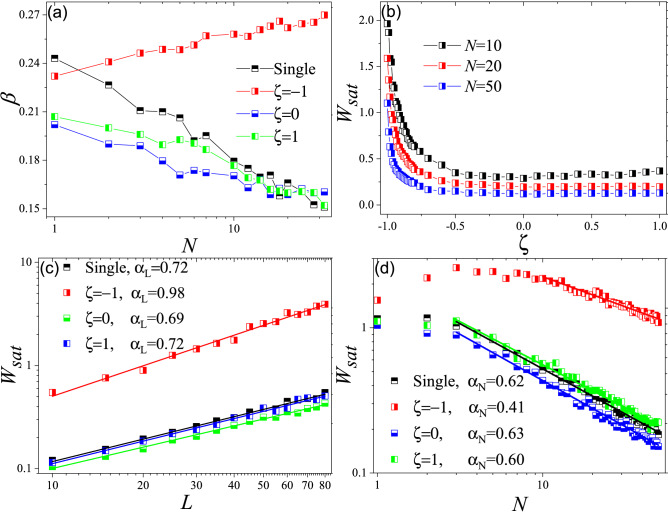
(**a**) Growth exponent,$\beta$ versus *N* for the single population model and two interactive populations with different values for $\zeta$. As this figure shows, *N* decreases *beta* only for non-competitive populations ($\zeta \ge 0$). (**b**) $W_{sat}$ versus $\zeta$ for $N=10$. This figure shows that for $\zeta =-1$ $W_{sat}$ is much larger that other values of $\zeta$ which means that for competitive populations, invasion front might be much rough. (**c**) $W_{sat}$ versus *L*. The slop of *log*/*log* diagram gives us the roughness exponent. (**d**) $W_{sat}$ versus *N*. For $\zeta =-1$ we have $\alpha _N=0.40$ and for $\zeta \ge 0$, we have $\alpha _N=0.61 \pm 0.02$.

We first obtain $\beta$ for different populations and as Fig. 5a shows, it decreases with *N* for non-competitive populations. However, for competitive populations, $\beta$ increases by *N*. To study the morphology of interface at steady state, we choose the saturated value of interface width, $W_{sat}$, which is the value *W* approaches in long times., and analyze its behavior versus *L*, *N* and $\zeta$. We tried to determine whether the interface follows any scaling behavior and then see how $\zeta$ affects the corresponding exponents. As Fig. 5b shows, $W_{sat}$ for $\zeta =-1$ is larger than other cases, which indicates that invasion front of competitive populations is rougher. Later we studied the behavior of $W_{sat}$ versus *L* and found the corresponding exponent, $\alpha _L$ (or simply $\alpha$ which is the roughness exponent). As Fig. 5c shows, for noncompetitive populations ($\zeta \ge 0$) $\alpha _L=0.70\pm 0.02$ but for competitive ones ($\zeta =-1$) we have $\alpha _L =0.98$. Finally, we studied the effect of *N* on $W_{sat}$. Interestingly, as Fig. 5d shows, competition ($\zeta =-1$) not only leads to higher interface roughness, the associated roughness also is less sensitive to *N*. While for $\zeta =-1$ we have $\alpha _N=0.41$, for $\zeta \ge 0$ we have $\alpha _N= 0.61\pm 0.02$.

These results reveal that invasion front morphology and its velocity and fluctuations depend on both interspecific interactions and environmental barriers intensity. These results reveal that the geometry of invasion front in competitive populations has significantly different behavior in response to stress. Thus, theoretically, one might be able to estimate clonal interactions by looking at the geometry of the invasion front. In competitive populations, each population cancels the effort of the counterpart population to occupy a new unit. When the intensity of barriers is low, it is more likely for one of the populations to degrade the barrier and occupy a new unit. However, as the intensity increases, such random events become less and less likely, leading to a significant decrease in invasion velocity. It should be noted that adding spatial heterogeneity to barrier resistance can increase the roughness of invasion front^30^.

## Discussion

Understanding tumor invasion through mathematical modeling and in vivo or in vitro studies has significantly increased our understanding of underlying mechanisms. In a now-classic example, it was suggested that duplication rate and diffusion rate of tumor cells determine tumor invasion velocity^47^. Since then, more parameters have been identified and taken to account to understand tumor invasion^48^. Yet, a lot has been remained to be understood about how the interplay between clonal interactions and environmental stress regulate tumor invasion. The fact that geometry of tumor plays a role in patient outcome, puts additional stress on the understanding of the behavior of invasion front.

The physical structure of the tumor environment provides a physical barrier against migration and cancer cells need to degrade the ECM to invade^9,11^. As such, we translate the stress to the physical barrier with the intensity of *N*. Here we considered the physical barrier as a limiting factor that prohibits further growth and cells need to degrade it. We found how interaction significantly regulates invasion velocity. Our results suggest that cooperation plays a crucial role in cells’ ability to overcome such a barrier. This conclusion is conceptually in line with other results on the relation between clonal interactions and environmental stress, such as nutrient shortage. We recently showed that once tumor cells individually acquire the ability to induce angiogenesis (angiogenic switch), they may not be able to grow larger until they cooperatively induce further angiogenesis^49^.

The geometry of the invasion front has been used to understand and predict tumor outcome^33^. As a growing interface, scaling analysis has been used to characterize the geometry of invasion front in different studies^30,34,36^. Most of these analyses have concentrated on how environmental features and cellular phenotype and activities such as duplication or dispersal affect the geometry of invasion front^31,34,36^, omitting the direct role of clonal interactions. In this model, two species can simultaneously occupy the same unit. Thus, they do not compete over limited space and their interactions are limited to invasion front. This assumption allows us to highlight the role of interactions on invasion front behavior (adding spatial exclusion makes the problem significantly challenging as we have studied in upcoming work). Here we showed that clonal interactions can single-handedly regulate invasion velocity and the geometry of the invasion front.

Irregularity of invasion front is associated with of tumor invasive behavior^32,33^, however, the reason behind this association has not been understood yet. Our results here show that local competition between individual cells can lead to irregular invasion front. On the other hand, the relation between poor clinical outcome and clonal diversity is well-established^18–24^. Thus, irregular geometry is not the cause of adverse clinical outcomes. Instead, both the irregular geometry and adverse outcome are results of clonal diversity and competition.

## Summary

Motivated by clonal interactions and environmental barriers that tumor cells experience, we developed a model to study how interspecific interactions and environmental stresses together regulate invasion. In 1D, we found the Langevin equation for invasion front and quantified the dependency of velocity and diffusion constant on the intensity of environmental barriers and the nature of interactions. It turned out that for single-species case, the invasion velocity depends on *N* as $V_f \propto N ^{-\gamma _V}$ with $\gamma _V=1.0$ and for the diffusion constant for invasion front, we have $D_f \propto N^{\gamma _D}$ with $\gamma _D=2.0$. Also, competitive populations are more vulnerable to environmental stress and their invasion velocity falls faster in response to *N* with $\gamma _V=1.80 \pm 0.04$. Diffusion constant for interactive populations ($\zeta \ne 0$) was generally larger and less sensitive to *N* compared to non-interactive populations ($\zeta =0$ or single population model). For the 2D case, the averaged invasion front (*H*) follows a Langevin equation which depends on *N* similar to 1D. The geometry of the invasion front exhibits scaling behavior. For $\zeta =-1$, we found that *N* increases $\beta$. The behavior of $W_{sat}$ versus *N*, *L* and $\zeta$ was obtained and it turned out that competition not only leads to more rough interfaces, but it also makes those interfaces resistant to environmental stresses. These findings deepen our understanding of the invasion of interactive species and may have applications to understanding tumor clonal interactions during the invasion.

It should be mentioned that real-world invasions, even those in highly controlled environments, are inherently complex processes. When it comes to cancer, invasion is highly regulated at different levels. Phenotypic plasticity, paracrine interactions with immune cells and fluctuating stresses are only a few relevant examples of huge number of processes that participate in the complex process of invasion. The goal of this work, has not been to deny other biologically relevant factors. Instead, we have tried to concentrate on the role of population level interactions and how they can change invasion front properties by simplifying different aspects.
